# Supplementary material for: Empirical methods for controlling false positives and estimating confidence in ChIP-Seq peaks
Source: BMC Bioinformatics. 2008 Dec 5;9:523. doi: 10.1186/1471-2105-9-523 (PMC2628906; doi:10.1186/1471-2105-9-523)
Supplement: Additional File 1 — A variety of html documents from the USeq web site detailing the available applications, their best usage, output file type descriptions, command line menus, etc. [file 1471-2105-9-523-S1.zip › USeqUserGuides/JavaDoc.zip!/JavaDoc/overview-tree.html]

Class Hierarchy


|  |  |  |
| --- | --- | --- |
| **Overview** \| Package \| Class \| Use \| **Tree** \| **Deprecated** \| **Index** \| **Help** | |  |
| PREV   NEXT | **FRAMES**    **NO FRAMES**     **All Classes** |  |


---


## Hierarchy For All Packages

**Package Hierarchies:**: bioroot, bioroot.antibody, bioroot.oligo, bioroot.parsers, bioroot.plasmid, bioroot.strain, cgar, cgar.beans, edu.utah.seq.analysis, edu.utah.seq.compression, edu.utah.seq.data, edu.utah.seq.methylation454, edu.utah.seq.misc, edu.utah.seq.parsers, expr, gata.aligner, gata.geneGlyphs, gata.main, gata.menu, gata.plotter, igb.util, meme, selex, trans.anno, trans.bkgrnd, trans.cel, trans.graphics, trans.main, trans.misc, trans.qc, trans.roc, trans.tpmap, util.amazon, util.apps, util.apps.gump, util.bio.annotation, util.bio.calc, util.bio.cluster, util.bio.converters, util.bio.digest, util.bio.parsers, util.bio.parsers.gff, util.bio.seq, util.bio.wrappers, util.bio.xamer, util.gen, util.xml

---

## Class Hierarchy

- java.lang.Object
  - util.bio.parsers.**ADFDataBaseSplitter**- trans.graphics.**AggregatePlots**- util.bio.converters.**AlignedBaseCounter**- gata.aligner.**AlignerPreferences** (implements java.io.Serializable)- gata.aligner.**Alignment** (implements java.lang.Comparable<T>, java.io.Serializable)- util.bio.calc.**Alignment**- gata.aligner.**AlignParams** (implements java.io.Serializable)- edu.utah.seq.methylation454.**Amplicon**- cgar.beans.**AnalysisBean**- cgar.beans.**AnalysisGroupBean**- cgar.beans.**AnalysisProtocolBean**- cgar.beans.**AnalysisTypeBean**- gata.main.**AnnoSpecParams**- trans.anno.**AnnotateRegions**- trans.anno.**AnnotateRegionsWithGeneList**- gata.geneGlyphs.**Annotation**- bioroot.antibody.**AntibodyPrep**- util.apps.**ArrayPrinter**- trans.cel.**AverageACels**- trans.misc.**AverageDoubles**- trans.cel.**AveragePCels**- trans.bkgrnd.**BackGroundOligo** (implements java.io.Serializable)- trans.misc.**Bar2Gr**- edu.utah.seq.parsers.**BarParser**- util.bio.parsers.**BaseCount**- edu.utah.seq.methylation454.**BaseRead**- trans.misc.**Bed2Gr**- trans.misc.**BestWindowScoreExtractor**- trans.roc.**Bin**- util.gen.**Bin**- util.bio.seq.**BinaryBooleanReaderWriter**- trans.main.**BindingPeak** (implements java.io.Serializable)- trans.anno.**BindingRegion** (implements java.lang.Comparable<T>)- trans.anno.**BindingRegionComparator** (implements java.util.Comparator<T>)- trans.main.**BindingRegionGraphPrinter**- trans.anno.**BindingRegionsToGff3**- trans.anno.**BindingRegionsToIntervals**- util.bio.wrappers.**BL2Seq**- util.bio.wrappers.**BL2SeqHit**- gata.aligner.**Blast**- util.bio.wrappers.**Blast**- util.bio.parsers.**BlastCompompositeGenerator**- trans.tpmap.**BlastFilterResult**- bioroot.**BlastMatcher** (implements java.lang.Comparable<T>)- util.bio.parsers.**BlastParser**- util.bio.wrappers.**BlastResult**- trans.graphics.**Box**- gata.aligner.**BrowseButtonAction** (implements java.awt.event.ActionListener)- trans.bkgrnd.**CalcBkgrndRatios**- trans.misc.**CalcGCAndTransform**- trans.misc.**CalcGCRatiosFromIntervals**- trans.bkgrnd.**CalculateBackgroundStats**- trans.misc.**CalculateGeneExtensions**- util.bio.parsers.**Cap3Parser**- trans.cel.**CelFileConverter**- trans.qc.**CelFileQualityControl**- trans.qc.**CelFileStats**- trans.cel.**CelLine** (implements java.lang.Comparable<T>)- bioroot.**CellTypeBean**- trans.cel.**CelMapper**- trans.graphics.**CelMasker**- trans.cel.**CelPHistogram**- trans.cel.**CelProcessor**- trans.cel.**ChromosomeIndex**- trans.main.**ChromSet**- util.bio.cluster.**Cluster** (implements java.io.Serializable)- trans.tpmap.**CollapseTPMap**- trans.graphics.**CombineXYSeries**- edu.utah.seq.data.**ComparatorEnrichedRegionPosition** (implements java.util.Comparator<T>)- edu.utah.seq.data.**ComparatorEnrichedRegionScore** (implements java.util.Comparator<T>)- edu.utah.seq.data.**ComparatorInfoPoint** (implements java.util.Comparator<T>)- trans.cel.**ComparatorMultiSetQuantile** (implements java.util.Comparator<T>)- edu.utah.seq.data.**ComparatorPointPosition** (implements java.util.Comparator<T>)- trans.cel.**ComparatorQuantile** (implements java.util.Comparator<T>)- edu.utah.seq.data.**ComparatorSmoothingWindowPosition** (implements java.util.Comparator<T>)- edu.utah.seq.data.**ComparatorSmoothingWindowScore** (implements java.util.Comparator<T>)- edu.utah.seq.data.**ComparatorSmoothingWindowTwoScore** (implements java.util.Comparator<T>)- java.awt.Component (implements java.awt.image.ImageObserver, java.awt.MenuContainer, java.io.Serializable)
                                                                                                                                                              - java.awt.Container
                                                                                                                                                                - javax.swing.JComponent (implements java.io.Serializable)
                                                                                                                                                                  - javax.swing.JPanel (implements javax.accessibility.Accessible)
                                                                                                                                                                    - gata.aligner.**AlignerInputPanel**- gata.plotter.**AlignPanel**- util.bio.cluster.**ClusterDrawPanel**- gata.menu.**FileInputPanel**- gata.geneGlyphs.**GlyphPanel**- util.apps.gump.**GumpApplicationPanel** (implements java.awt.event.ComponentListener)- util.apps.gump.**GumpToolPanel** (implements java.awt.event.ActionListener)- trans.graphics.**IntervalDrawPanel**- gata.plotter.**MultiAlignPanel**- trans.graphics.**RankedSetDrawPanel**- gata.menu.**SaveImagePanel** (implements java.awt.event.ActionListener)- util.apps.**ScatterDrawPanel**- trans.graphics.**VirtualCelPanel**
                                                                                                                                                                    - javax.swing.JScrollPane (implements javax.accessibility.Accessible, javax.swing.ScrollPaneConstants)
                                                                                                                                                                      - util.apps.gump.**GumpResultsScrollPane**
                                                                                                                                                                  - java.awt.Window (implements javax.accessibility.Accessible)
                                                                                                                                                                    - java.awt.Frame (implements java.awt.MenuContainer)
                                                                                                                                                                      - javax.swing.JFrame (implements javax.accessibility.Accessible, javax.swing.RootPaneContainer, javax.swing.WindowConstants)
                                                                                                                                                                        - gata.aligner.**AlignerInputForm**- trans.qc.**ChartFrame**- util.bio.cluster.**ClusterDrawFrame**- gata.plotter.**Console**- gata.menu.**FileInputForm**- gata.menu.**GATAFrame**- util.apps.gump.**GumpDrawFrame**- util.apps.gump.**GumpResults**- trans.graphics.**IntervalDrawFrame**- trans.graphics.**RankedSetDrawFrame**- gata.menu.**SaveImageForm**- util.apps.**ScatterDrawFrame**- gata.main.**SimpleTextWindow**- util.gen.**TextFrame**- gata.main.**TextWindow**- gata.plotter.**ToolsFrame**- trans.graphics.**VirtualCelFrame**
                                                                                                                                                              - edu.utah.seq.compression.**CompressFasta**- gata.plotter.**ConservedSeqs**- bioroot.**ContactBean**- trans.roc.**ControlGene**- trans.cel.**ControlStats** (implements java.io.Serializable)- trans.misc.**ConvertAgilentData**- trans.misc.**ConvertBars2TPMapCel**- util.bio.seq.**ConvertFasta2GCBoolean**- util.bio.seq.**ConvertFasta2RealSeqBoolean**- trans.misc.**ConvertGeoData**- trans.misc.**ConvertNimblegenNDF2TPMap**- trans.misc.**ConvertNimblegenPAIR2Cela**- trans.tpmap.**ConvertTPMapsToFeatures**- trans.cel.**ConvertTVSToCelps**- util.bio.annotation.**Coordinate** (implements java.lang.Comparable<T>)
                                                                                                                                                                                            - util.bio.annotation.**ScoredCoordinate**
                                                                                                                                                                                            - trans.qc.**CoordinateExtractor1lq**- util.apps.**Correlate**- trans.misc.**CorrelateGrFiles**- expr.**CorrelationMaps**- expr.**CorrelationWindow**- edu.utah.seq.methylation454.**CpG**- bioroot.**CronArchive**- util.gen.**CubicSpline**- edu.utah.seq.methylation454.**DataLine** (implements java.lang.Comparable<T>)- bioroot.**DBUtil**- edu.utah.seq.analysis.**DefinedRegionScanSeqs**- bioroot.**DeleteMeNow**- trans.misc.**Delme**- util.apps.**Delme2**- gata.aligner.**DelmeAligner**- cgar.**DelmeTest**- trans.qc.**DestypeIntensityCalculator**- edu.utah.seq.data.**DividePointData**- trans.anno.**DmelCGNameCoordinatePrinter**- util.bio.parsers.gff.**DmelRel4Extractor**- util.bio.parsers.**DrosGeneCollFileParser**- edu.utah.seq.parsers.**ElandExportLine**- edu.utah.seq.parsers.**ElandMultiParser**- edu.utah.seq.parsers.**ElandParser**- edu.utah.seq.parsers.**ElandSequenceParser**- util.gen.**Email**- edu.utah.seq.data.**EnrichedRegion** (implements java.io.Serializable)- edu.utah.seq.analysis.**EnrichedRegionMaker**- util.bio.digest.**Enzyme** (implements java.lang.Comparable<T>)- util.bio.annotation.**ExonIntron** (implements java.lang.Comparable<T>, java.io.Serializable)- trans.roc.**ExonScoreExtractor**- util.bio.annotation.**ExportExons**- util.bio.annotation.**ExportIntergenicRegions**- trans.main.**ExportIntervalData**- util.bio.annotation.**ExportIntronicRegions**- expr.**ExpressedGene** (implements java.lang.Comparable<T>)- expr.**ExpressionScores2CoordinatesViaGFF**- util.bio.parsers.gff.**ExtractGffFeatures**- trans.roc.**ExtractNonOverlappingWindows**- trans.roc.**ExtractSaveSgrScores**- trans.roc.**ExtractScoresForRoc**- bioroot.parsers.**ExtractWords**- util.bio.parsers.**Fasta** (implements java.lang.Comparable<T>)- trans.main.**FDRVsQValues**- trans.main.**FDRWindowConverter**- bioroot.**Feature** (implements java.lang.Comparable<T>)- util.bio.parsers.**FetchGenomicSequences**- util.bio.seq.**FetchIndexedSequence**- util.bio.parsers.**FetchSeqsQualsByName**- expr.**FileCrossFilter**- gata.aligner.**FileFocusListener** (implements java.awt.event.FocusListener)- util.apps.**FileJoiner**- expr.**FileMatchJoiner**- util.gen.**FileSize** (implements java.lang.Comparable<T>)- util.apps.**FileSplitter**- util.apps.**FileTrimmer**- util.apps.**FilterAgilentProbeFile**- edu.utah.seq.data.**FilterPointData**- trans.tpmap.**FilterTPMapByRegions**- trans.anno.**FindNeighboringGenes**- trans.main.**FindSubBindingRegions**- util.apps.**Flash**- util.bio.parsers.gff.**GadFlyGffExtractor**- gata.aligner.**GATAligner**- gata.main.**GATAParams**- gata.main.**GATAPlotter**- gata.main.**GATAUtil**- trans.misc.**GCRatio** (implements java.lang.Comparable<T>)- bioroot.**GeneBean**- util.apps.**GeneCentricCNVParser**- util.bio.annotation.**GeneGroup** (implements java.lang.Comparable<T>, java.io.Serializable)- util.apps.**GeneNameCounter**- util.bio.annotation.**GeneRep** (implements java.io.Serializable)- gata.geneGlyphs.**GeneRepGlyph**- util.bio.annotation.**GenericFeature** (implements java.lang.Comparable<T>)- gata.geneGlyphs.**GenericGlyph**- javax.servlet.GenericServlet (implements java.io.Serializable, javax.servlet.Servlet, javax.servlet.ServletConfig)
                                                                                                                                                                                                                                                                                                                                                      - javax.servlet.http.HttpServlet (implements java.io.Serializable)
                                                                                                                                                                                                                                                                                                                                                        - bioroot.**AccountManagement**- bioroot.antibody.**AntibodyBase**- bioroot.antibody.**AntibodyModify**- bioroot.antibody.**AntibodySpreadSheet**- bioroot.antibody.**AntibodyViewPreferences**- bioroot.**Archive**- bioroot.**CellType**- bioroot.**Contact**- bioroot.**Gene**- bioroot.**Login**- cgar.**Login**- bioroot.**Logout**- bioroot.**Marker**- bioroot.antibody.**NewAntibody**- bioroot.oligo.**NewOligo**- bioroot.plasmid.**NewPlasmid**- bioroot.strain.**NewStrain**- bioroot.oligo.**OligoBase**- bioroot.oligo.**OligoModify**- bioroot.oligo.**OligoSpreadSheet**- bioroot.oligo.**OligoUpload**- bioroot.oligo.**OligoViewPreferences**- bioroot.**Organism**- bioroot.plasmid.**PlasmidBase**- bioroot.plasmid.**PlasmidModify**- bioroot.plasmid.**PlasmidSpreadSheet**- bioroot.plasmid.**PlasmidUpload**- bioroot.plasmid.**PlasmidViewPreferences**- bioroot.**Search**- bioroot.strain.**StrainBase**- bioroot.strain.**StrainModify**- bioroot.strain.**StrainSpreadSheet**- bioroot.strain.**StrainUpload**- bioroot.strain.**StrainViewPreferences**- cgar.**TestInBioTools**- bioroot.**UserRegistration**
                                                                                                                                                                                                                                                                                                                                                      - cgar.beans.**GenomeBean**- util.bio.converters.**GenomeFASTASplitter**- trans.graphics.**GenomicRegion**- trans.graphics.**GenomicRegionGlyph**- trans.anno.**Gff2ToBindingRegions**- trans.anno.**Gff2ToIntervals**- util.bio.parsers.gff.**Gff3Feature** (implements java.lang.Cloneable, java.lang.Comparable<T>)- util.bio.parsers.gff.**Gff3Parser**- util.bio.parsers.gff.**GffFeature**- util.bio.parsers.gff.**GffParser**- trans.roc.**Gr**- trans.misc.**Gr2Bar**- trans.graphics.**Graph**- trans.roc.**GrComparator** (implements java.util.Comparator<T>)- trans.misc.**GrGraph**- util.apps.gump.**Gump**- edu.utah.seq.data.**HeatMapMaker**- edu.utah.seq.data.**HeatMapMakerPosNeg**- util.bio.cluster.**HierarchicalClustering**- util.gen.**Histogram**- igb.util.**HistogramSgrOrGrFile**- util.apps.**HouseBean**- util.gen.**HTML**- bioroot.**IdNameMatch**- util.bio.seq.**IndexedSequence** (implements java.lang.Comparable<T>)- util.bio.seq.**IndexFastas**- util.bio.seq.**IndexRegions**- trans.misc.**InefficientPseudoMedian**- edu.utah.seq.data.**Info** (implements java.io.Serializable)- trans.tpmap.**IntensityFeature** (implements java.lang.Comparable<T>, java.io.Serializable)- trans.main.**IntensityPrinter**- trans.anno.**IntersectBindingPeaks**- trans.anno.**IntersectKeyWithRegions**- util.apps.**IntersectLists**- trans.anno.**IntersectRegions**- trans.main.**Interval** (implements java.lang.Comparable<T>, java.io.Serializable)- trans.main.**IntervalComparator** (implements java.util.Comparator<T>)- trans.main.**IntervalFilter**- trans.main.**IntervalGFFPrinter**- trans.main.**IntervalGraphPrinter**- trans.main.**IntervalMaker**- trans.graphics.**IntervalPlotter**- trans.main.**IntervalReportPrinter**- util.gen.**IO**- util.gen.**Job**- edu.utah.seq.parsers.**JohnsonToTag**- util.apps.**JQSub**- bioroot.**LabGroup**- cgar.beans.**LabGroupBean**- cgar.**LabGroupUserType**- util.gen.**LinearRegression**- trans.main.**LoadChipSetIntervalOligoInfo**- trans.bkgrnd.**LoadedOligo** (implements java.lang.Comparable<T>)- trans.main.**LoadIntervalOligoInfo**- gata.aligner.**LocalAlignment** (implements java.io.Serializable)- bioroot.**LoginBean**- cgar.beans.**LoginBean**- util.bio.seq.**LowerCase2Xs**- trans.main.**MakeAllWindows**- util.apps.**MakeBioTools**- trans.cel.**MakeChromosomeSets**- util.bio.seq.**MakeSpliceJunctionFasta**- util.bio.seq.**MakeTranscriptome**- edu.utah.seq.misc.**MakeUSeq**- trans.bkgrnd.**MapControlIntensities**- trans.tpmap.**MapFeature** (implements java.lang.Comparable<T>, java.io.Serializable)- trans.tpmap.**MapSplitter**- bioroot.**MarkerBean**- util.bio.seq.**MaskExonsInFastaFiles**- util.gen.**MD5Crypt**- meme.**MemeMotif**- meme.**MemeParser**- meme.**MemeR**- meme.**MemeResults**- util.apps.**MergeColumns**- util.bio.seq.**MergeFastas**- trans.anno.**MergeRegions**- trans.main.**MergeWindowArrays**- java.security.MessageDigestSpi
                                                                                                                                                                                                                                                                                                                                                                                                                                                                                                                    - java.security.MessageDigest
                                                                                                                                                                                                                                                                                                                                                                                                                                                                                                                      - util.gen.**MD5** (implements java.lang.Cloneable)
                                                                                                                                                                                                                                                                                                                                                                                                                                                                                                                    - trans.roc.**MethodTester**- edu.utah.seq.methylation454.**MethylationParser**- expr.**MiRNA**- expr.**MiRNATarget**- util.gen.**Misc**- meme.**MotifHit** (implements java.lang.Comparable<T>)- meme.**MotifScanner**- meme.**MotifSearchResult** (implements java.lang.Comparable<T>)- util.bio.parsers.**MultiFastaParser**- trans.cel.**MultiSetQuantile** (implements java.io.Serializable)- trans.main.**MultiSGRPrinter**- trans.main.**MultiWindowIntervalMaker**- trans.tpmap.**MummerMapper**- util.bio.digest.**NEBParser**- expr.**NormalizeAgilentData**- util.bio.calc.**NucleicAcid**- util.gen.**Num**- trans.main.**Oligo** (implements java.io.Serializable)- trans.tpmap.**OligoFlipper**- trans.main.**OligoIntensityPrinter**- bioroot.plasmid.**OligoMatch**- util.bio.seq.**OligoTiler**- bioroot.oligo.**OligoUploadBean**- util.apps.gump.**Option**- bioroot.**OrganismBean**- bioroot.**Organization**- trans.main.**OverlapCounter**- expr.**ParseAgilentData**- util.bio.parsers.**ParseDecodeGenotypeData**- expr.**ParseManipulateColumnDataForGeneSifter**- expr.**ParseMiRNATable**- util.apps.**ParseMLSHTML**- trans.roc.**ParsePatternedWindows**- trans.qc.**Parser1lq**- util.apps.**ParserForGff**- util.bio.seq.**ParseSeqsFromUCSCGeneTable**- trans.roc.**ParseSgrsForParticularRegions**- trans.roc.**ParseSgrsForParticularScores**- util.bio.parsers.gff.**ParseWormBaseGffToUCSC**- expr.**ParseWormMart**- util.xml.**ParseXMLFile**- util.gen.**Passwords**- bioroot.**PatternMatch**- trans.main.**PeakPicker**- edu.utah.seq.analysis.**PeakPicker**- util.gen.**PearsonCorrelation**- bioroot.plasmid.**PlasmidUploadBean**- trans.graphics.**PlotFloatArrays**- gata.main.**PlotterPreferences** (implements java.io.Serializable)- edu.utah.seq.data.**Point**
                                                                                                                                                                                                                                                                                                                                                                                                                                                                                                                                                                                                                        - edu.utah.seq.data.**InfoPoint**
                                                                                                                                                                                                                                                                                                                                                                                                                                                                                                                                                                                                                        - edu.utah.seq.data.**PointData**- trans.roc.**Positive**- trans.roc.**PositiveComparator** (implements java.util.Comparator<T>)- trans.main.**PosRegion** (implements java.lang.Comparable<T>)- util.bio.converters.**PossumToGFF**- bioroot.**Preference**
                                                                                                                                                                                                                                                                                                                                                                                                                                                                                                                                                                                                                                    - bioroot.strain.**StrainPreference**
                                                                                                                                                                                                                                                                                                                                                                                                                                                                                                                                                                                                                                    - trans.misc.**PrependColumn**- util.bio.wrappers.**Primer3Pick**- util.bio.wrappers.**Primer3Wrapper**- util.apps.**PrintSelectColumns**- cgar.beans.**ProcessedDataBean**- cgar.beans.**ProcessedDataTypeBean**- trans.roc.**PWMScoreWindows**- edu.utah.seq.analysis.**QCSeqs**- util.bio.parsers.**QualityFileParser**- trans.cel.**Quantile**- trans.cel.**QuantileNormalization**- trans.main.**RandomizeLabels**- trans.anno.**RandomRegions**- util.gen.**RankedFloatArray**- trans.graphics.**RankedSetAnalysis**- util.gen.**RankSample**- util.gen.**RankSampleIndexComparator** (implements java.util.Comparator<T>)- util.gen.**RankSampleValueComparator** (implements java.util.Comparator<T>)- cgar.beans.**RawDataBean**- edu.utah.seq.methylation454.**Read**- edu.utah.seq.methylation454.**ReadAlignParser**- util.gen.**ReadZip**- bioroot.**ReagentBean** (implements java.lang.Comparable<T>)
                                                                                                                                                                                                                                                                                                                                                                                                                                                                                                                                                                                                                                                                                  - bioroot.antibody.**AntibodyBean**- bioroot.oligo.**OligoBean**- bioroot.plasmid.**PlasmidBean**- bioroot.strain.**StrainBean**
                                                                                                                                                                                                                                                                                                                                                                                                                                                                                                                                                                                                                                                                                  - util.bio.digest.**REDigest**- edu.utah.seq.methylation454.**Reference**- util.bio.parsers.**RefSeqAnnotationParser**- trans.anno.**Region** (implements java.io.Serializable)- trans.anno.**RegionComparator** (implements java.util.Comparator<T>)- trans.misc.**Regions2Intervals**- util.apps.**RenameRWSResults**- bioroot.parsers.**RenameWords**- util.bio.converters.**RepeatMaskerToGFF**- trans.main.**ReplaceWindowScores**- util.bio.parsers.**RipInparanoidResults**- util.bio.parsers.**RipMGIOrthologyResults**- trans.roc.**RocWindow** (implements java.lang.Comparable<T>, java.io.Serializable)- trans.roc.**RocWindowScanner**- trans.cel.**RotateCelFile**- util.amazon.**S3CodeSamples**- util.amazon.**S3Manager**- trans.main.**SamChromosomeData**- edu.utah.seq.methylation454.**Sample**- gata.geneGlyphs.**ScaleBar**- trans.main.**ScanChip**- trans.main.**ScanChromosomes**- trans.main.**ScanChromosomesCNV**- trans.main.**ScanGenes**- edu.utah.seq.analysis.**ScanSeqs**- util.apps.**ScatterPlot**- util.apps.**ScoreChromosomes**- trans.main.**ScoredGene** (implements java.lang.Comparable<T>)- trans.misc.**ScoredRegionsFormatFixer**- trans.main.**ScoreIntervals**- trans.roc.**ScoreParsedBars**- util.apps.**ScoreSequences**- bioroot.**SearchBean**- bioroot.**SearchHit** (implements java.lang.Comparable<T>)- util.bio.wrappers.**Segment**- selex.**SelexParams**- selex.**SelexSeqParser**- util.bio.seq.**Seq**- selex.**SeqFiles**- selex.**SeqRead**- gata.aligner.**Seqs**- trans.main.**SetNumberIntervalMaker**- trans.roc.**Sgr** (implements java.io.Serializable)- trans.misc.**Sgr2Bar**- trans.roc.**SgrBacCounter**- trans.misc.**SgrComparator** (implements java.util.Comparator<T>)- util.bio.parsers.**ShiftPositionsInUCSCGeneLines**- util.bio.wrappers.**SimpleBlast**- edu.utah.seq.data.**Simulator**- gata.plotter.**Sliders**- util.apps.**SmoothData**- edu.utah.seq.data.**SmoothingWindow** (implements java.io.Serializable)- edu.utah.seq.data.**SmoothingWindowInfo** (implements java.io.Serializable)- bioroot.**Spammer**- util.gen.**SpearmanCorrelation**- cgar.beans.**SpeciesBean**- trans.cel.**SplineScalar**- trans.main.**SplitWindowArraysByChromosome**- util.gen.**SQL**- gata.plotter.**StartStop** (implements java.lang.Comparable<T>)- util.bio.annotation.**StartStop** (implements java.lang.Comparable<T>, java.io.Serializable)- trans.tpmap.**Stat1lq**- util.apps.**StatBarFiles**- trans.qc.**StatFlag**- trans.cel.**StatIntensityFeatureArray**- edu.utah.seq.methylation454.**StatMethylationData**- trans.tpmap.**StatTPMap**- trans.tpmap.**StatTPMapControlSeqs**- bioroot.strain.**StrainUploadBean**- expr.**StripZeros**- edu.utah.seq.data.**SubSamplePointData**- selex.**SubSeq**- util.apps.**SubtractOneFromEachColumn**- trans.anno.**SubtractRegions**- trans.main.**SubWindow** (implements java.io.Serializable)- util.gen.**Swing**- util.bio.parsers.**SynonymMatching**- trans.main.**T2**- trans.main.**T2Parameter**- bioroot.**TableHeading** (implements java.lang.Comparable<T>)- edu.utah.seq.parsers.**Tag2Point**- util.apps.**TASSignalChromFileSplitter**- trans.main.**TestScanChip**- util.apps.**TextParser**- trans.misc.**TextPValOrSig2Gr**- java.lang.Thread (implements java.lang.Runnable)
                                                                                                                                                                                                                                                                                                                                                                                                                                                                                                                                                                                                                                                                                                                                                                                                                                                              - gata.aligner.**AlignerThread**
                                                                                                                                                                                                                                                                                                                                                                                                                                                                                                                                                                                                                                                                                                                                                                                                                                                              - trans.tpmap.**TPMapDensityReducer**- trans.tpmap.**TPMapDuplicateFilter**- trans.tpmap.**TPMapFeature** (implements java.io.Serializable)- trans.tpmap.**TPMapLine** (implements java.lang.Comparable<T>)- trans.tpmap.**TPMapOligoBlastFilter**- trans.tpmap.**TPMapProcessor**- trans.tpmap.**TPMapSort**- trans.tpmap.**TPMapStripHighLowGC**- util.bio.annotation.**Transcript** (implements java.io.Serializable)- trans.misc.**TransformRatios**- util.bio.annotation.**TransGroup** (implements java.io.Serializable)- gata.geneGlyphs.**TransGrpGlyph**- util.bio.annotation.**Translation** (implements java.io.Serializable)- util.bio.parsers.**UCSCGeneLine**- util.bio.parsers.**UCSCGeneLineComparator** (implements java.util.Comparator<T>)- util.bio.parsers.**UCSCGeneLineComparatorScore** (implements java.util.Comparator<T>)- util.bio.parsers.**UCSCGeneModelTableReader**- bioroot.antibody.**UseDilution**- bioroot.**UserBean**- cgar.beans.**UserBean**- bioroot.**UserRegistrationBean**- cgar.beans.**UserTypeBean**- bioroot.**Util**- cgar.**Util**- trans.misc.**Util**- meme.**UtilMeme**- selex.**UtilSelex**- trans.graphics.**VirtualCel**- cgar.beans.**VisibilityBean**- trans.misc.**Wig2Bar**- trans.main.**WilcoxonRankSumTest**- trans.main.**WilcoxonSample** (implements java.lang.Comparable<T>)- trans.main.**WilcoxonSamplePair** (implements java.lang.Comparable<T>)- trans.main.**WilcoxonSignedRankTest**- trans.main.**Window** (implements java.lang.Comparable<T>, java.io.Serializable)- trans.main.**WindowBlockMaker**- trans.main.**WindowBlockMakerTwoColor**- trans.main.**WindowComparator** (implements java.util.Comparator<T>)- trans.misc.**WindowHistogram**- trans.tpmap.**WindowMaker**- igb.util.**Windows2HeatMapSgr**- util.apps.**WindowScoreSmoother**- util.bio.xamer.**Xamer**- util.bio.xamer.**XamerCounter**- util.gen.**XML**- util.apps.**ZipAndDelete**

---


|  |  |  |
| --- | --- | --- |
| **Overview** \| Package \| Class \| Use \| **Tree** \| **Deprecated** \| **Index** \| **Help** | |  |
| PREV   NEXT | **FRAMES**    **NO FRAMES**     **All Classes** |  |


---
